# A quantum dots dual immunochromatography strip for differential detection of high and low virulence ASFV antibodies

**DOI:** 10.1128/spectrum.02551-25

**Published:** 2025-11-28

**Authors:** Shuai Zhang, Yuzhu Zuo, Huixia Fan, Jing Ma, Yunhuan Zhao, Yan Li, Chuanwen Wang, Jinghui Fan

**Affiliations:** 1 College of Veterinary Medicine, Hebei Agricultural University 74562, Baoding, Hebei, China; 2 College of Information Science and Technology, Hebei Agricultural University 74562, Baoding, Hebei, China; National Chung Hsing University, Taiwan, Province of China

**Keywords:** African swine fever virus, immunochromatographic strip, quantum dots

## Abstract

African swine fever (ASF), caused by African swine fever virus (ASFV), is a highly contagious disease that infects both domestic and wild pigs and poses a significant threat to the global pig industry. Given the absence of vaccines and effective treatments, the development of rapid and precise serological diagnostic methods is critical for the prevention and control of ASFV. However, traditional enzyme-linked immunosorbent assay (ELISA) is unsuitable for point-of-care testing (POCT). Immunochromatographic strip (ICS) offers portability but suffers from low sensitivity and an inability to differentiate between high virulence wild-type ASFV and low virulence ASFVΔCD2v infections. Consequently, there is an urgent need to develop a rapid, sensitive, and accurate ICS that can distinguish between wild-type ASFV and ASFVΔCD2v infection for ASFV control. In this study, we developed a novel quantum dot (QDs) dual ICS using p54 and CD2v proteins as test 1 line (T1 line) and test 2 line (T2 line) capture antigens, and QDs as the tracer for specific ASFV antibody detection. The QDs dual ICS had no cross-reactivity with other swine disease antibodies, could detect ASFV antibody-positive serum diluted to 1:10^6^, and exhibited excellent repeatability, reproducibility, and stability. A total of 589 clinical samples were analyzed using QDs dual ICS. Compared to commercial ELISA kits, T1 line achieved 99.66% accuracy, 100% sensitivity, and 99.62% specificity, and T2 line achieved 100% accuracy, sensitivity, and specificity. It offers a new technical method for POCT of ASFV antibodies and differentiation between wild-type ASFV and ASFVΔCD2v infection.

**IMPORTANCE:**

The concurrent prevalence of both high-virulence wild-type ASFV and low-virulence ASFVΔCD2v has increased the pressure on the prevention and control of ASFV. Based on the p54 and CD2v proteins of ASFV, a highly sensitive and specific quantum dots (QDs) dual immunochromatography strip (ICS) was developed using QDs conjugated with SPA as a tracer. The developed novel QDs dual ICS can rapidly detect ASFV antibodies and differentiate from high-virulence wild-type ASFV or low-virulence ASFVΔCD2v infections. This novel QDs dual ICS could be used for the serological differential diagnosis and epidemiology study of ASFV.

## INTRODUCTION

ASF is an acute and highly virulent infectious disease caused by the ASFV that primarily affects pigs. The disease poses significant economic and social challenges and a serious threat to the global pig farming industry (1, 2). ASF is listed as a notifiable disease by the World Organization for Animal Health (WOAH) (2). ASFV was first discovered in Kenya in 1921 and then spread to Europe in 1957. Subsequently, it spread to regions such as the Americas and Russia (3–5). In 2018, high virulence genotype II wild-type ASFV was introduced in China and rapidly spread to all 31 provinces, triggering a large-scale ASF epidemic (6, 7). Within a year of the introduction of ASFV, hundreds of millions of pigs have been culled, either due to deaths from ASFV infection or preventive slaughters to control the spread of ASFV. As the largest pork producer and consumer, ASFV has had a major impact on the economy of China and poses a serious threat to food security (8). Currently, ASF remains endemic in more than 50 countries across Africa, Asia, and Europe, resulting in significant economic losses to the global pork industry (9, 10). After the outbreak, the evolution of ASFV accelerated, leading to gradual weakening of its virulence. ASFV gradually transforms from the most acute and acute forms to subacute and chronic forms. In some pig farms in China and other countries affected by ASFV, naturally occurring low virulence genotype II ASFV with deletion or mutation of the EP402R gene (ASFVΔCD2v), resulting in non-expression of CD2v protein, has emerged (1, 11–15). Chronic or asymptomatic ASF caused by ASFVΔCD2v is highly contagious. Pigs infected with ASFVΔCD2v intermittently excrete ASFV and infect susceptible pigs through direct or indirect contact, thus becoming potential latent sources of infection (16–18). Compared with wild-type ASFV, ASFVΔCD2v exhibits less obvious clinical symptoms and is challenging to detect using pathogen diagnostic methods such as polymerase chain reaction (PCR) in clinical settings (19). This facilitates the silent spread of ASFVΔCD2v within pig farms, thereby posing significant challenges to ASFV prevention and control. In 2021, naturally occurring low virulence genotype I ASFV strains with non-expression of CD2v protein have also emerged, which can cause chronic infection accompanied by atypical signs such as necrotic skin lesions and joint swelling (20). Subsequently, high virulence and lethality natural recombinant viruses of genotype I and II ASFV strains have been detected in Jiangsu, Henan, and Inner Mongolia (21). The co-circulation of these diverse ASFV strains further complicates the prevention and control of ASF in China.

ASFV is a large enveloped double-stranded DNA (dsDNA) virus belonging to the Asfarviridae family and is classified as a member of the nucleocytoplasmic large DNA viruses (NCLDVs) (22). The genome of ASFV is 170–190 kb in length and can encode over 150 proteins. The p54 protein, encoded by the E183L gene, is a key antigenic protein and an important structural protein in ASFV. p54 protein is expressed during the early phases of ASFV infection. It plays a crucial role in inducing antiviral antibodies during ASFV infection and exhibits strong immunogenicity, antigenicity, and conservation (23–25). Consequently, it is considered the most suitable diagnostic antigen and is widely used as a target for detecting antibodies against ASFV infection. The CD2v protein encoded by EP402R is a glycosylated structural protein located on the outer envelope of ASFV (26, 27). It plays a crucial role in facilitating the spread of viral particles within the host and is essential for the development of ASFV vaccines and disease-prevention strategies (28, 29). Domestic and international studies have revealed that ASFV strains featuring EP402R gene deletions or mutations result in a significant decrease in both the virulence and pathogenicity (1, 12–15). Therefore, establishing a detection method that can be used to distinguish between ASFVΔCD2v and wild-type ASFV infection is of great significance for the prevention and control of ASFV.

ASFV has a history of more than a century as a top threat to the pig farming industry. Scholars have conducted extensive research and development of ASF vaccines. Currently, the types of ASF vaccines under investigation include inactivated, attenuated live, and genetically engineered vaccines. However, because of its large genomic structure and intricate immune evasion mechanisms, no safe and effective vaccine has been developed for the prevention and control of ASFV, apart from two attenuated live vaccines that have been commercialized in Vietnam (30, 31). Currently, the prevention and control of disease outbreaks rely primarily on stringent biosecurity protocols and advanced diagnostic techniques. Rapid and intuitive detection methods are important for the prevention and control of ASFV infection. At present, methods for ASFV detection include virus isolation, hemadsorption (HAD) test, PCR, and real-time PCR (32, 33). However, these methods are not only time-consuming but also require sophisticated equipment and specialized personnel, thereby limiting their application in POCT. In addition, pigs infected with ASFVΔCD2v exhibit subclinical, chronic, or asymptomatic infections. Following infection, the ASFV titer in surviving pigs gradually decreases, while antibody levels progressively increase and persist (34). This reduction in ASFV titer may lower the detection rate of ASFV nucleic acids. As carriers of ASFV, these pigs excrete ASFV and infect susceptible animals through direct and indirect contact, thereby facilitating the sustained transmission of ASFV within pig farms. Therefore, monitoring ASFV antibodies is crucial to assess the infection status of pigs. Currently, the most commonly used ASFV antibody detection methods are ELISA and ICS (10, 19, 32, 35). However, the ELISA method is complex, time-consuming, and requires specialized personnel to be performed in well-equipped laboratories. This limitation forces small-scale farms, which often lack diagnostic facilities, to rely on third-party diagnostic services, thereby increasing both turnaround time and cost of detection. Therefore, the development of an ASFV antibody detection method suitable for POCT is critically important. ICS is widely used in POCT because of its simple operation, minimal requirement for specialized personnel and precision instruments, and visual reading of results in the field (36, 37). However, the colloidal gold ICS (CG ICS) commonly employed for POCT exhibits relatively low sensitivity. Although currently published ICS based on fluorescent nanomaterials has achieved improved sensitivity, they uniformly rely on a single test line targeting proteins such as p54, p72, and pp62 (17, 38, 39). Distinguishing between ASFVΔCD2v and wild-type ASFV infections would require additional tests, such as a separate ICS or ELISA targeting the CD2v protein. Therefore, these existing ICS are incapable of distinguishing between ASFVΔCD2v and wild-type ASFV infections in a single reaction. Consequently, there is an urgent need to develop a simple, highly sensitive antibody detection method suitable for POCT that can distinguish between ASFV△CD2v and wild-type ASFV infections.

Among fluorescent nanomaterials, quantum dots (QDs) exhibit excellent optical properties exhibit superior optical properties characterized by narrow emission peaks, broad absorption spectra, size-tunable luminescence, excellent photostability, and high quantum yields (40–42). As tracers, QDs can significantly improve the sensitivity of ICS and serve as ideal fluorescent probes (43). In this study, we developed a QDs dual ICS based on the p54 and CD2v proteins for the first time. These QDs dual ICS can simultaneously detect antibodies against both p54 and CD2v proteins in the field, enabling differentiation between ASFVΔCD2v and wild-type ASFV infections. It offers a technical method for monitoring, preventing, and controlling ASFV.

## MATERIALS AND METHODS

### Materials, reagents, and sera

DH5α and BL21(DE3) competent cells and the pET-32a vector were maintained in our laboratory. The QDs (core composition: ZnCdSe/ZnS; emission wavelength: 625 ± 5 nm; mass concentration: 5 mg/mL) were purchased from Wuhan Jiayuan Quantum Dots Co., Ltd. (Wuhan, China). 2-Morpholinoethanesulfonic acid monohydrate (MES) and 1-ethyl-(3-dimethylaminopropyl) carbodiimide hydrochloride (EDC) were purchased from Beijing Soleibao Technology Co. Ltd. (Beijing, China). Blood filter pads (Cat.No. GF8), conjugate pads (Cat.No. Ahlstrom 8964), NC membranes (Cat.No. Sartorius CN95), absorbent pads (Cat.No. H5015), and PVC plates (Cat.No. DB-6) were purchased from Shanghai Jieyi Biotechnology Co., Ltd. (Shanghai, China). Wild-type ASFV and ASFVΔCD2v antibody-positive and ASFV-negative standard sera were generously provided by Qingdao Lijian Biotechnology Co., LTD (Qingdao, China). Serum samples of porcine circovirus type 2 (PCV2^+^), porcine pseudorabies virus (PRV^+^), classical swine fever virus (CSFV^+^), and porcine reproductive and respiratory syndrome virus (PRRSV^+^) were obtained from our laboratory. From October 2021 to February 2025, 589 clinical samples were collected from pig farms suspected of ASFV infection in Hebei Province, Shanxi Province, Shandong Province, Jiangsu Province, Heilongjiang Province, and Inner Mongolia Autonomous Region. The animal owners had verbally agreed to the participation of the animals in the research. This study did not involve any invasive procedures, such as infection challenges or dissections of pigs, during its execution. The detection of ASFV antibodies in this study was performed solely on serum samples collected by animal owners for routine serological surveillance of other diseases. Given that no additional animal-based experiments were planned, the study proceeded based on the oral informed consent provided by the animal owners for their animals’ participation in the research.

### Expression, purification, and western blotting of p54 and CD2v proteins

The p54 and CD2v proteins were expressed and purified according to previously reported methods (44). In brief, the E183L and EP402R gene sequences of ASFV Pig/HLJ/2018 (GenBank: MK333180.1) were optimized, synthesized, and cloned into the pET-32a vector by Sangon Biological Engineering Co., Ltd. (Shanghai, China). These recombinant plasmids were further transformed into BL21 (DE3) competent cells and the induction conditions were optimized. Bacteria were collected and ultrasonically crushed (SCIENTZ, Ningbo, China). The p54 and CD2v recombinant proteins were analyzed by M5 Prestained Protein Ladder (catalog number: MF212, Mei5bio, Beijing, China) and SDS-PAGE and purified using a His-labeled protein purification kit (CWBIO, Jiangsu, China). Protein concentrations were determined using the BCA Protein Concentration Determination Kit (Beyotime, Shanghai, China). Endotoxins were removed using a ToxinEraser Endotoxin Removal Kit (Genscript, Nanjing, China) and detected using a ToxinSensor Chromogenic LAL Endotoxin Assay Kit (GenScript).

Purified proteins were separated by SDS-PAGE and transferred onto polyvinylidene fluoride (PVDF) membranes. The PVDF membranes were sealed in Tris-buffered saline-Tween (TBST) solution containing 5% skim milk powder. Wild-type ASFV and ASFVΔCD2v positive and ASFV negative standard sera were used as the primary antibodies, and horseradish peroxidase-conjugated rabbit anti-pig IgG(H + L) (Biodragon, Beijing, China) was used as the secondary antibody. The results of western blotting were analyzed using Western Protein Marker (catalog number: G2086, Servicebio, Wuhan, China), an eECL Western Blot Kit (CWBIO), and an Odyssey Fc infrared imaging system (LI-COR, Lincoln, NE, USA).

### Conjugation of staphylococcal protein A and QDs

Under dark conditions, 100 µL of 1 µg/µL QDs was added to 100 µL of MES solution (0.02 M, pH 7.0), followed by addition of 2 µL of EDC solution (10 mg/mL). The mixture was incubated (37°C and 200 rpm) for 20 min. After centrifugation at 12,000 rpm for 15 min at 4°C, the precipitate was resuspended in 100 µL MES solution (0.02 M, pH 7.0). Next, 20 µg of SPA was added and incubated (37°C, 200 rpm) for 3 h. Finally, 4 µL of 10% (wt/vol) BSA solution (pH 7.0) was added and incubated (37°C, 200 rpm) for 1 h. After resuspension in 100 µL PBS solution (0.02 M, pH 7.4), the QDs conjugated with SPA (QDs-SPA) were obtained. After preparation, the QDs-SPA was identified and characterized to ensure conjugation.

### Assembly and reaction principle of the QDs dual ICS

**Fig 1:**
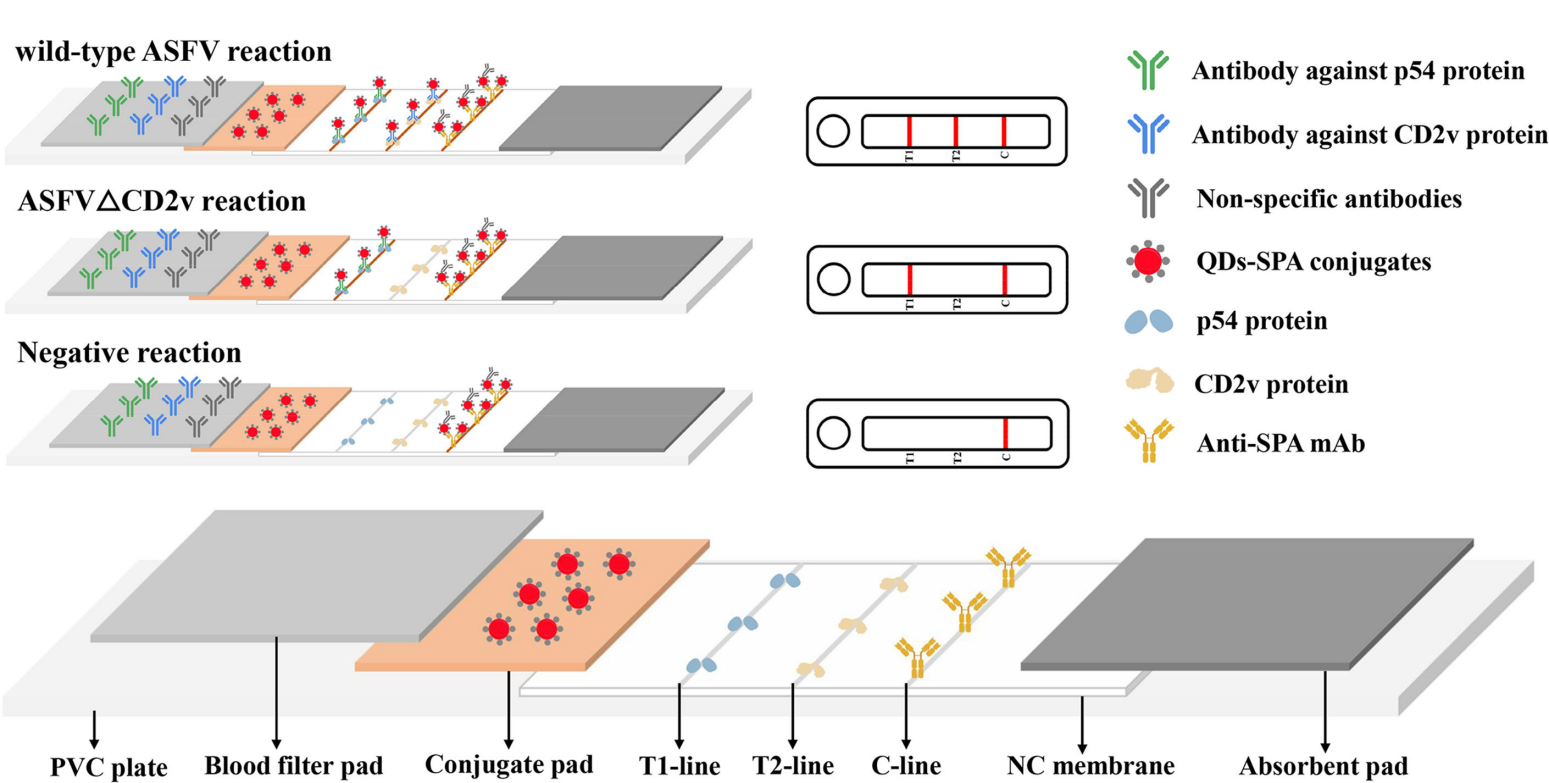
Schematic of the proposed QDs dual ICS for ASFV antibody detection.

The QDs dual ICS consists of four parts: a blood filter pad, a conjugate pad, an NC membrane, and an absorbent pad (Fig. 1). The conjugate pad was prepared by soaking in Tris-HCl (0.05M, containing 0.5% BSA, 0.8% sucrose, 0.1% PEG-1500, and 0.5% Tween-20 for 20 min) and in QDs-SPA solution for 3 h, followed by drying in a 37°C oven for 1 h. The NC membrane was prepared by coating p54 protein, CD2v protein, and monoclonal anti-SPA mAb (Bersee, Beijing, China) on the NC membrane of the T1 line, T2 line, and the control line (C line), respectively, and drying in an oven at 37°C for 1 h. The prepared blood filter pad, conjugate pad, NC membrane, and absorbent pad were sequentially overlapped by 2 mm and adhered to a PVC plate to assemble the QDs dual ICS.

The QDs dual ICS reaction principle relies on the specific binding between antibodies and antigens. Diluted blood samples were separated into serum samples using a blood filter pad and then migrated to the conjugate pad via capillary action. If antibodies against wild-type ASFV (p54 and CD2v protein antibodies: positive) are present, these antibodies and other IgG antibodies bind to QDs-SPA to form immune complexes (QDs-SPA-p54, QDs-SPA-CD2v, and QDs-SPA-IgG). These complexes were captured on the T1, T2, and C lines, resulting in three visible lines. If antibodies against ASFVΔCD2v (p54 protein antibodies: positive, CD2v protein antibodies: negative) are present, only p54 and other IgG antibodies bind to QDs-SPA, forming immune complexes (QDs-SPA-p54 and QDs-SPA-IgG) that are captured on the T1 and C lines, resulting in two visible lines. If the serum lacks ASFV antibodies (p54 and CD2v protein antibodies: negative), only other IgG antibodies bind to QDs-SPA and are captured on the C line, resulting in one visible line. If the C line had no color, the test results were invalid.

### Optimization of the preparation conditions for QDs dual ICS

#### Optimization of the pH value for QDs conjugated with SPA

To determine the optimal pH value, wild-type ASFV standard serum was detected by QDs dual ICS prepared by conjugating QDs with SPA in 0.02 M MES buffer with pH values of 6, 6.5, 7, 7.5, and 8. The fluorescence intensities of the T1 line (FI_T1_), T2 line (FI_T2_), and C line (FI_C_) were measured using a fluorescence immunoassay analyzer to determine the optimal pH value.

#### Optimization of the concentration of SPA conjugated with QDs

To determine the optimal concentration of SPA conjugated with QDs, wild-type ASFV standard serum was detected by QDs dual ICS prepared by conjugating QDs with 15, 20, 25, 30, and 35 µg SPA. The optimal SPA concentration was determined by measuring FI_T1_, FI_T2_, and FI_C_.

#### Optimization of the coated concentration for the T1, T2, and C line

The p54 protein was diluted to 0.5, 1, 1.5, 2, and 2.5 µg/µL using 0.01 M Tris(hydroxymethyl)aminomethane hydrochloride (Tris HCl) buffer (pH 7.4, 5% sucrose), and subsequently coated onto the T1 line. Wild-type ASFV antibody-positive standard serum and ASFV antibody-negative standard serum were detected using the QDs dual ICS. The optimal concentration of p54 protein for coating the T1 line was determined by measuring FI_T1_ and calculating the positive and negative FI ratios (P/N ratio). Subsequently, the optimal coating concentrations for T2 and C lines were determined using an identical procedure.

#### Optimization of the sample dilution

Wild-type ASFV antibody-positive standard serum and ASFV antibody-negative standard serum were diluted with the sample diluent at 1:50, 1:100, 1:200, and 1:400 and detected by the QDs dual ICS. The FI was measured, and the P/N ratio was calculated to determine the optimal sample dilution.

#### Optimization of the immunoreaction time

Under the optimal preparation conditions for QDs dual ICS, wild-type ASFV antibody positive standard serum and ASFV antibody negative standard serum were detected by the QDs dual ICS. The optimal immunoreaction time was determined by measuring the FI at different immunoreaction times (5, 10, 15, 20, and 25 min) and by calculating the P/N ratio.

### Performance analysis of QDs dual ICS

#### Analytical sensitivity of QDs dual ICS

The wild-type ASFV antibody-positive standard serum was diluted 1:10^1^, 1:10^2^, 1:10^3^, 1:10^4^, 1:10^5^, and 1:10^6^, and then detected by the QDs dual ICS. Analytical sensitivity was evaluated by measuring the FI.

#### Analytical specificity of QDs dual ICS

The serum positive for PCV2, PRV, CSFV, PRRSV, wild-type ASFV, and ASFVΔCD2v and negative serum of ASFV were diluted at 1:10^2^ and then detected by the QDs dual ICS. FI was measured to evaluate the analytical specificity of the QDs dual ICS.

#### Repeatability and reproducibility of QDs dual ICS

To evaluate the repeatability of the QDs dual ICS, three QDs dual ICS of the same batch were used to detect serum samples of different states to obtain within-batch coefficients of variation (CV = (SD/mean) × 100%). Additionally, to assess the reproducibility of the QDs dual ICS, three different batches of QDs dual ICS were used to detect serum samples of different states to obtain the inter-batch CVs in three independent laboratories.

#### Stability of QDs dual ICS

To confirm the stability of the dual ICS QDs, the same batch of QDs dual ICS was stored at 4, 25, and 37°C for 9 months. During this time, the QDs dual ICS was periodically utilized to detect serum samples to obtain the CVs.

#### Detection of clinical samples

To verify the applicability of the QDs dual ICS in clinical sample detection, 589 clinical samples were analyzed using QDs dual ICS and commercial ASFV antibody ELISA kits in parallel. Accuracy was calculated based on the detection results.

### Statistical analysis

All experiments were independently replicated at least thrice. All data were statistically analyzed using Origin 8.0 (OriginLab, Northampton, MA, USA) and SPSS 27.0 (IBM Corp, Armonk, NY, USA). The results of the analysis are expressed as mean ± SD.

## RESULTS

### Expression, purification, and western blotting of p54 and CD2v proteins

**Fig 2:**
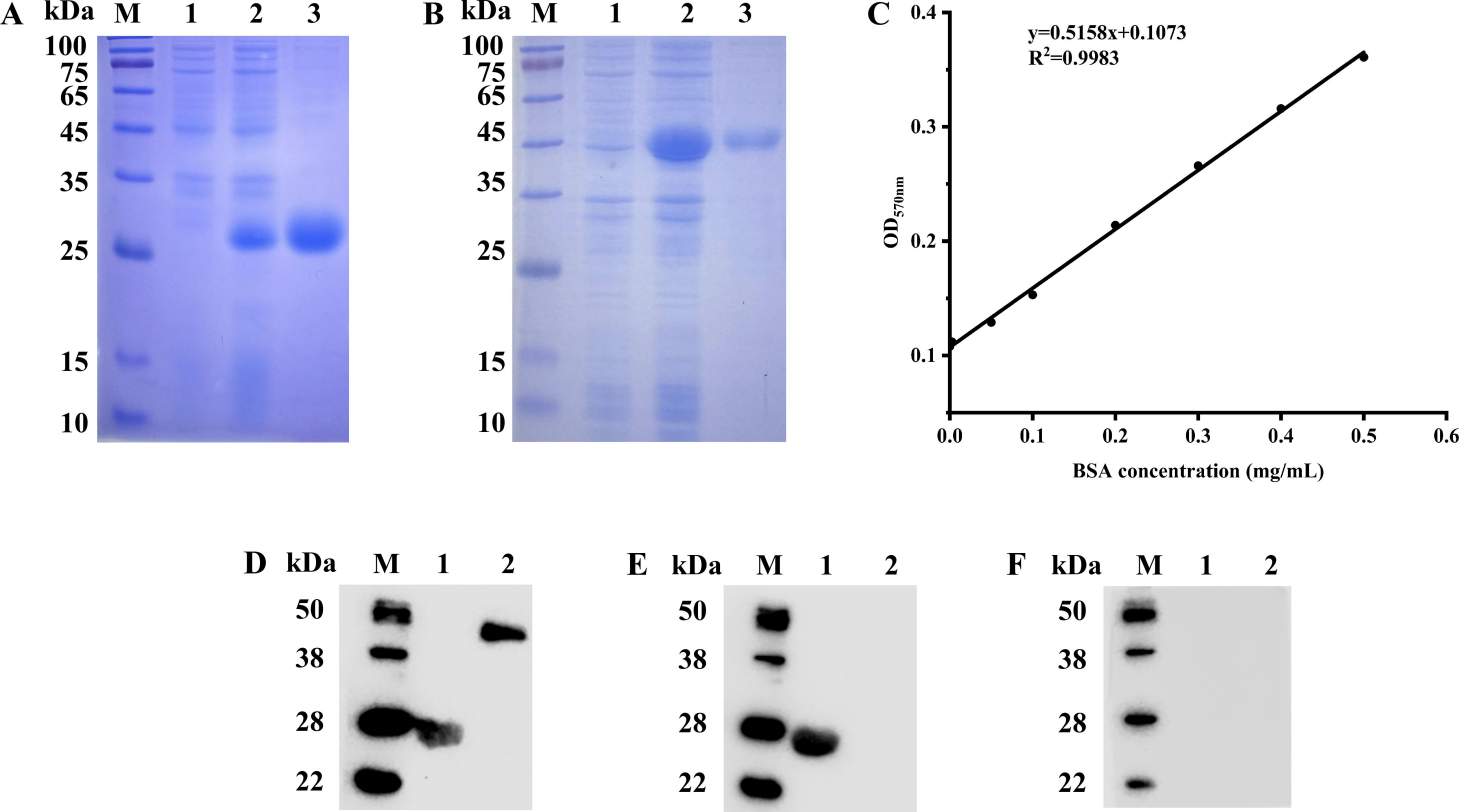
Expression, purification, concentration determination, and western blotting of p54 and CD2v proteins. Analysis of the p54 (**A**) and CD2v (**B**) proteins by the SDS-PAGE. Lane M, protein marker. Lane 1, Whole bacteria before induction. Lane 2, Whole bacteria after induction. Lane 3, purified protein. (**C**) Concentration determination of p54 and CD2v proteins by a BCA protein assay kit. Western blotting analysis of p54 and CD2v proteins with wild-type ASFV (**D**) and ASFVΔCD2v (**E**) positive and ASFV negative (**F**) standard sera. Lane M, protein marker. Lane 1, p54 protein. Lane 2, CD2v protein.

After induction with IPTG, the recombinant proteins p54 (Fig. 2A) and CD2v (Fig. 2B) were successfully expressed in *Escherichia coli* (optimal expression conditions: 0.1 mM IPTG, 37°C for 5 h). SDS-PAGE analysis confirmed that the molecular weights of the p54 and CD2v recombinant proteins were 27.8 kDa and 39.8 kDa, respectively, and the purification yield was satisfactory. Treatment with the endotoxin removal kit resulted in endotoxin levels below the detection limit. The concentrations of the p54 and CD2v recombinant proteins were determined to be 1.250 mg/mL (OD_570nm_: 0.752) and 1.324 mg/mL (OD_570nm_: 0.790), respectively, using a BCA protein assay kit (Fig. 2C). Further western blot analysis using wild-type ASFV (Fig. 2D) and ASFVΔCD2v (Fig. 2E) positive and ASFV negative (Fig. 2F) standard sera showed that the recombinant protein p54 specifically reacted with both wild-type ASFV and ASFVΔCD2v positive standard sera, but not with ASFV negative serum. In contrast, the recombinant protein CD2v only reacted specifically with wild-type ASFV-positive serum and did not react with the ASFVΔCD2v positive or ASFV negative standard sera. These results indicate that both recombinant proteins exhibited good immunoreactivity and specificity.

### Characterization of QDs and QDs-SPA conjugates

**Fig 3:**
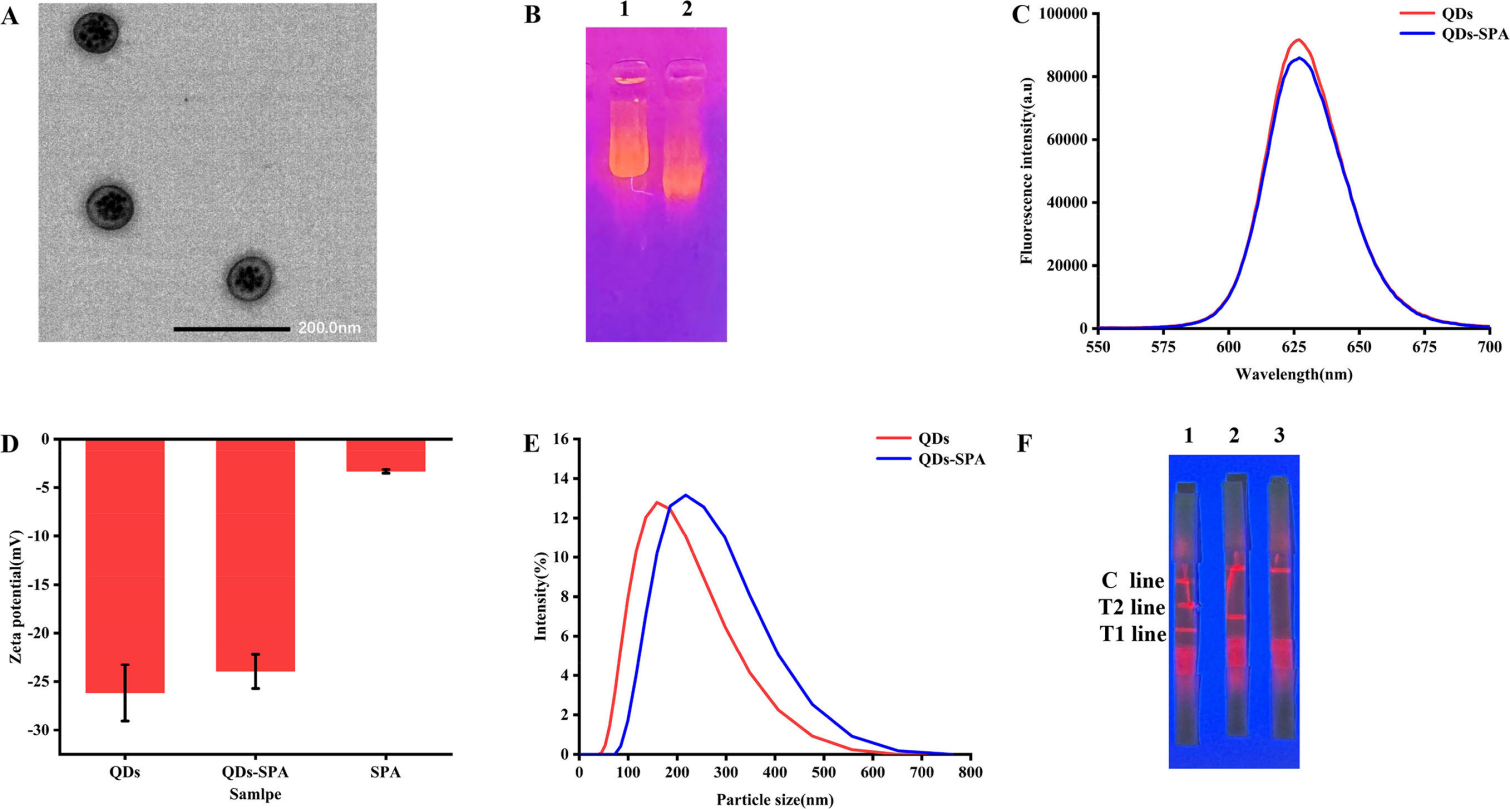
Characterization of QDs and QDs-SPA conjugates. (**A**) TEM of QDs. (**B**) Agarose gel electrophoresis of QDs and QDs-SPA. Lane 1, QDs-SPA. Lane 2, QDs. (**C**) Fluorescence emission spectra of QDs and QDs-SPA. (**D**) Zeta potentials of QDs, QDs-SPA, and SPA. (**E**) Particle size of QDs and QDs-SPA. (**F**) Original bioactivity of QDs-SPA. Lane 1, wild-type ASFV positive standard sera. Lane 2, ASFVΔCD2v positive standard sera. Lane 3, ASFV negative standard sera.

The successful conjugation of the QDs with SPA and the original bioactivity of the QDs-SPA were verified through a series of characterization techniques. First, the QDs were placed on a carbon-coated copper grid and analyzed using transmission electron microscopy (TEM) (JEOL, Japan). The observations indicated that the QDs displayed an irregular but predominantly spherical shape at the edges and were distributed inside the microspheres (Fig. 3A). Second, agarose gel electrophoresis analysis revealed that under identical experimental conditions, the migration rate of the QDs-SPA conjugates was markedly slower than that of the QDs alone, which might be due to the conjugation of QDs and SPA (Fig. 3B). Then, QDs and QDs-SPA conjugates were added, and fluorescence spectra analysis was performed using a fluorescence spectrophotometer (Shimadzu, Japan). In the emission wavelength range of 550–700 nm, the maximum emission peak was observed at 627 nm (Fig. 3C). Compared to the QDs, the QDs-SPA conjugates retained a high level of fluorescence intensity. At the same concentration, the fluorescence intensity retention rate of QDs-SPA conjugates was 93.78%. This result not only demonstrated that the QDs-SPA conjugates maintained fluorescence performance during the conjugation process but also provided preliminary evidence of the successful conjugation of QDs with SPA. The zeta potentials of SPA, QDs, and QDs-SPA conjugates were measured using a zeta potential analyzer (Bettersize, China). The results showed that the Zeta potential of SPA was −3.33 mV, the Zeta potential of QDs was −26.15 mV, and the Zeta potential of the QDs-SPA conjugates was −23.94 mV (Fig. 3D). These findings indicate that the QDs exhibit excellent stability. Even though the stability of the QDs-SPA conjugates was slightly reduced compared to that of the QDs, they still maintained satisfactory stability. In addition, the particle sizes of the QDs and QDs-SPA conjugates were characterized by dynamic light scattering (DLS; Bettersize, China), the particle size of QDs-SPA increased from 154.16 nm to 250.56 nm compared with those of the QDs, indicating the successful conjugation of the QDs with SPA (Fig. 3E). The original bioactivity of the prepared QDs-SPA conjugates was validated using immunochromatography. The results demonstrated that for wild-type ASFV positive serum, distinct bands appeared at the T1, T2, and C lines; for ASFVΔCD2v positive serum, bands were observed at the T1 and C lines; and for ASFV negative serum, a band was only present at the C line (Fig. 3F). These findings confirmed that the QDs-SPA conjugates exhibited original bioactivity and were suitable for diagnostic applications. In conclusion, QDs were successfully conjugated with SPA. The QDs-SPA conjugates exhibited excellent fluorescence performance and colloidal stability following conjugation.

### Optimization of the preparation conditions for QDs dual ICS

#### Optimization of the pH value for QDs conjugated with SPA

**Fig 4:**
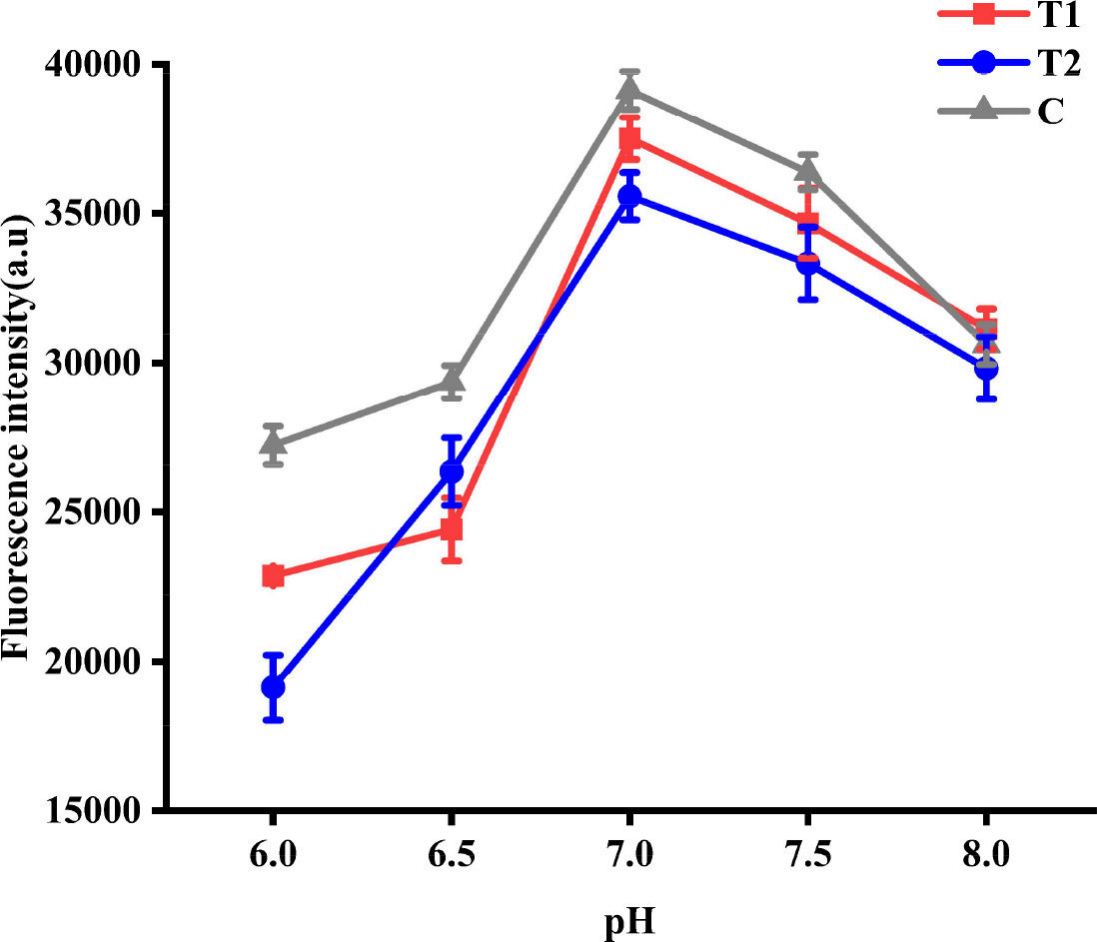
Optimization of the pH value for QDs conjugated with SPA. FI_T1_, FI_T2_, and FI_C_ at different pH values.

After activation with MES and EDC solutions, the precipitate of the activated QDs was resuspended in MES solution at pH values of 6, 6.5, 7, 7.5, and 8. An equal amount of SPA was added to prepare QDs with dual ICS. Subsequently, QDs with dual ICS prepared under different pH conditions were evaluated using wild-type ASFV standard serum. FI_T1_, FI_T2_, and FI_C_ levels were measured using a fluorescence immunoassay analyzer. The results showed that FI_T1_, FI_T2_, and FI_C_ reached their maximum values in the MES solution at pH 7.0 (Fig. 4). Therefore, the optimal pH for QDs conjugated with SPA was determined to be 7.0.

#### Optimization of the concentration of SPA conjugated with QDs

**Fig 5:**
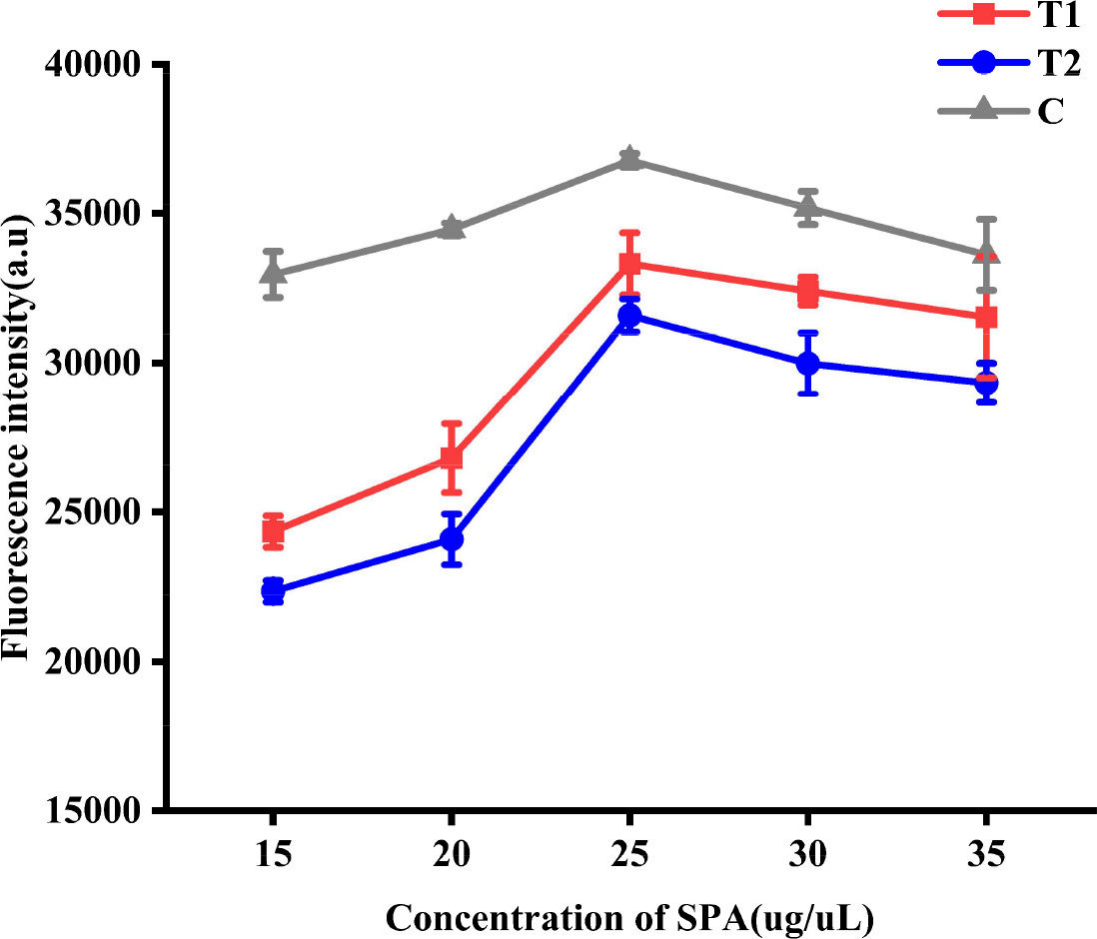
Optimization of the concentration of SPA conjugated with QDs. FI_T1_, FI_T2_, and FI_C_ at different concentration of SPA conjugated with QDs.

After activation with MES and EDC solutions and resuspension with MES at an optimal pH value of 7.0. Different amounts of SPA (15, 20, 25, 30, and 35 µg) were added to prepare the QDs dual ICS. Wild-type ASFV standard serum was detected using QDs dual ICS, and FI_T1_, FI_T2_, and FI_C_ were measured using a fluorescence immunoassay analyzer. The results demonstrated that, as the SPA concentration increased, FI_T1_, FI_T2_, and FI_C_ exhibited an upward trend, peaking at an addition of 25 µg, and subsequently stabilized and gradually declined (Fig. 5). Therefore, 0.25 µg/µl was considered the optimal concentration of SPA conjugated with QDs.

#### Optimization of the coated concentration for the T1, T2, and C lines

**Fig 6:**
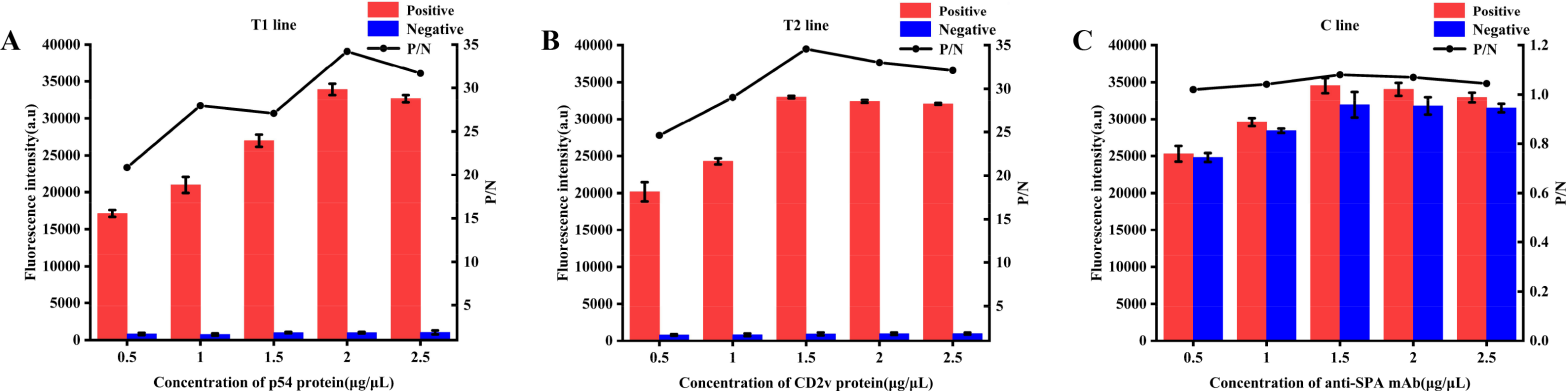
Optimization of the coated concentration for the T1, T2, and C line. FI_T1_ (**A**), FI_T2_ (**B**), and FI_C_ (**C**) at different coated concentration.

The optimization of the coated concentration for the T1, T2, and C lines showed that the FI of wild-type ASFV antibody-positive standard serum was the strongest and the P/N ratios reached their maximum values when the concentration of the coated p54 protein, CD2v protein, and anti-SPA mAb were 2 (Fig. 6A), 1.5 (Fig. 6B), and 1.5 mg/mL (Fig. 6C), respectively. Therefore, 2 (p54 protein), 1.5 (CD2v protein), and 1.5 mg/mL (anti-SPA mAb) were considered the optimal coated concentration for the T1, T2, and C line.

#### Optimization of the sample dilution

**Fig 7:**
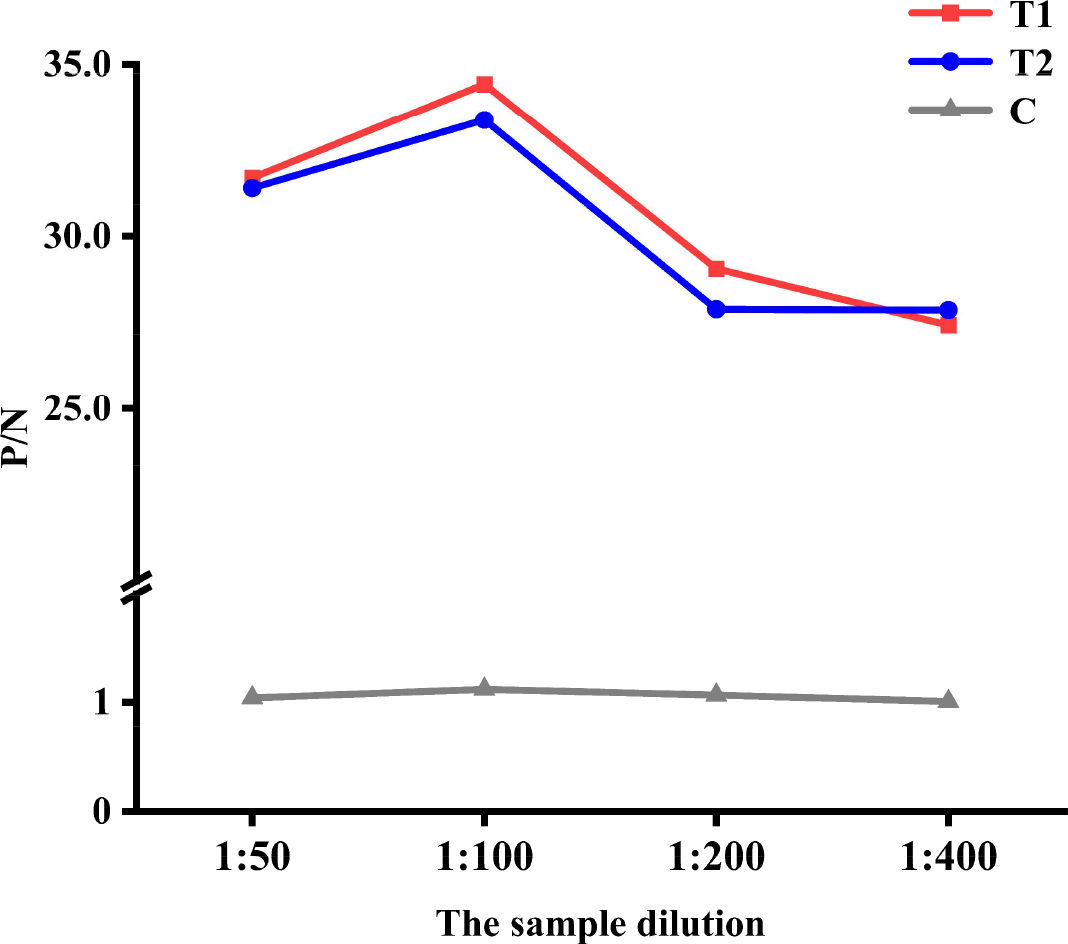
Optimization of the sample dilution. P/N ratio at different sample dilutions.

Different dilutions were used to optimize the sample dilution. The results showed that the P/N ratio reached a maximum when the wild-type ASFV antibody-positive standard serum and ASFV antibody-negative standard serum were diluted 1:100 (Fig. 7). Therefore, a 1:100 dilution was determined to be the optimal sample dilution.

#### Optimization of the immunoreaction time

**Fig 8:**
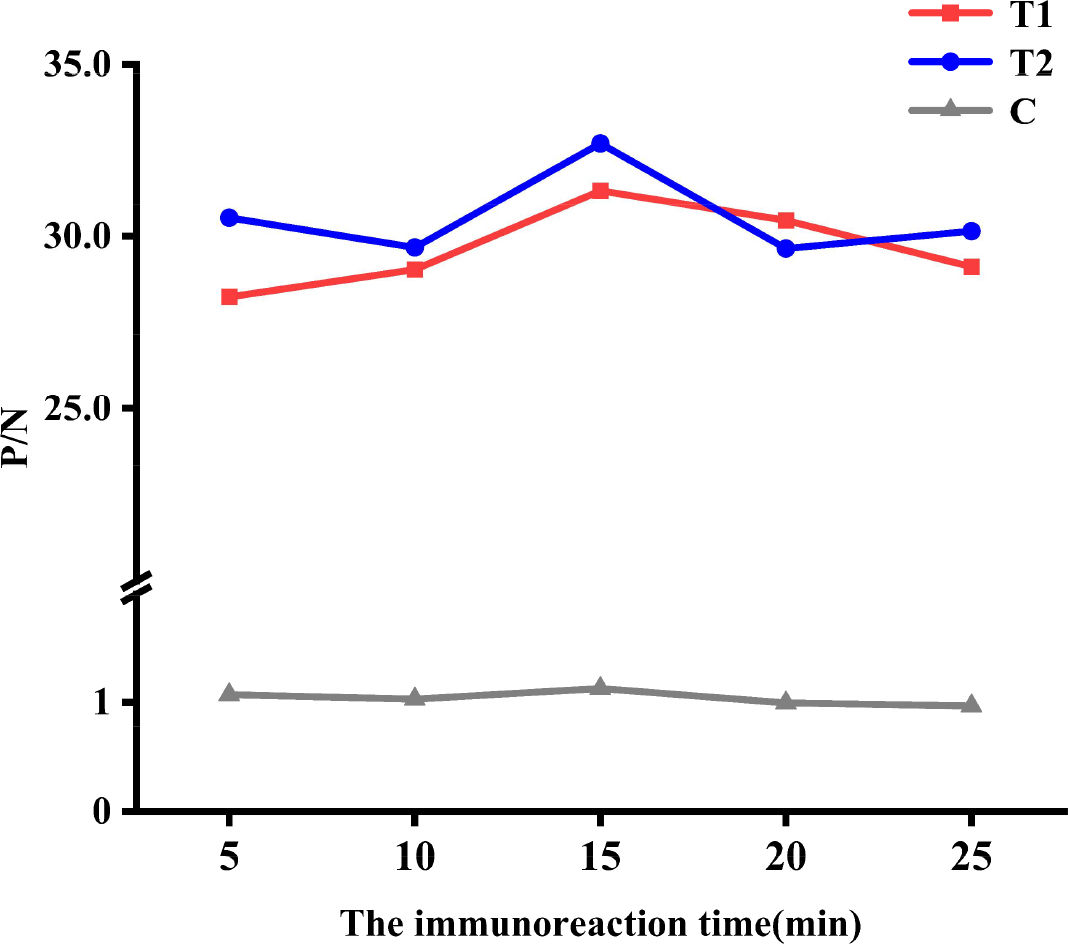
Optimization of the immunoreaction time. *P*/*N* ratio at different immunoreaction time.

The immunoreaction times were optimized as 5, 10, 15, 20, and 25 min. As the immunoreaction time increased, the FI of the wild-type ASFV antibody-positive standard serum gradually increased and subsequently began to decline after 15 min (Fig. 8). Notably, the P/N ratio reached its maximum after 15 min. Therefore, the optimal immunoreaction time was determined to be 15 min.

### Performance analysis of QDs dual ICS

#### Analytical sensitivity of QDs dual ICS

**Fig 9:**
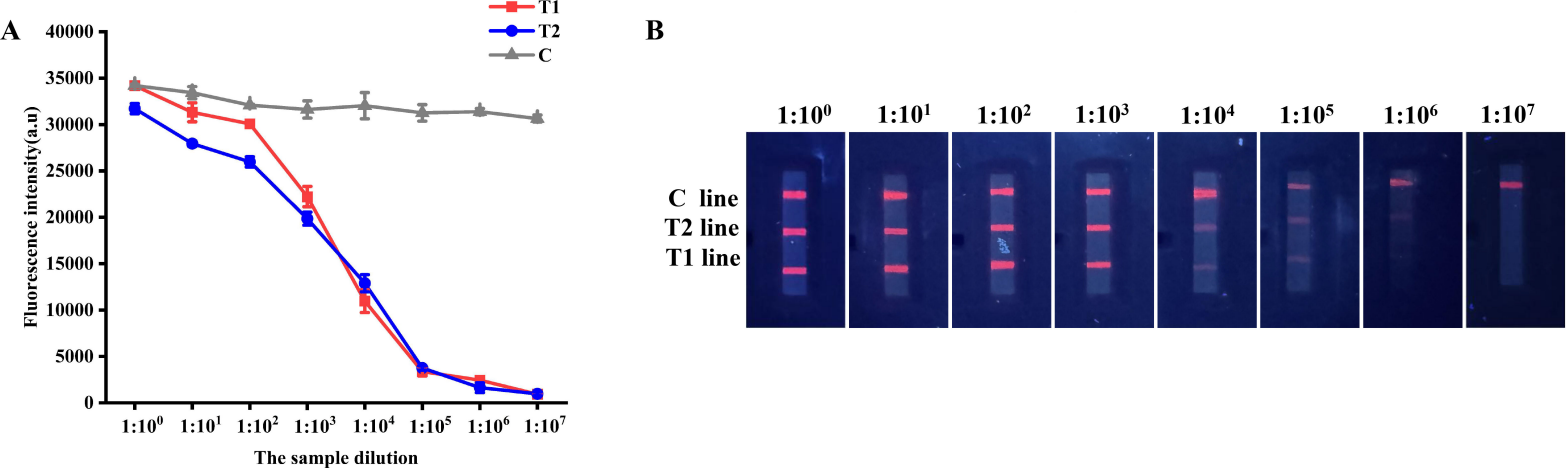
Analytical sensitivity of QDs dual ICS. (**A**) FI_T1_, FI_T2_, and FI_C_ at serial dilutions of wild-type ASFV antibody positive standard serum by fluorescence immunoassay analyzer. (**B**) Image of T1, T2, and C line at serial dilutions of wild-type ASFV antibody positive standard serum by portable UV lamp.

The analytical sensitivity of QDs dual ICS was evaluated using serial dilutions of wild-type ASFV antibody-positive standard serum. The results of QDs dual ICS detection showed that as the concentration of the serum sample decreased, FI_T1_ and FI_T2_ gradually diminished (Fig. 9A). Notably, distinct bands were visible to the naked eye at a dilution of 1:10^6^ (Fig. 9B). Therefore, the QDs dual ICS exhibited significant sensitivity.

#### Analytical specificity of QDs dual ICS

**Fig 10:**
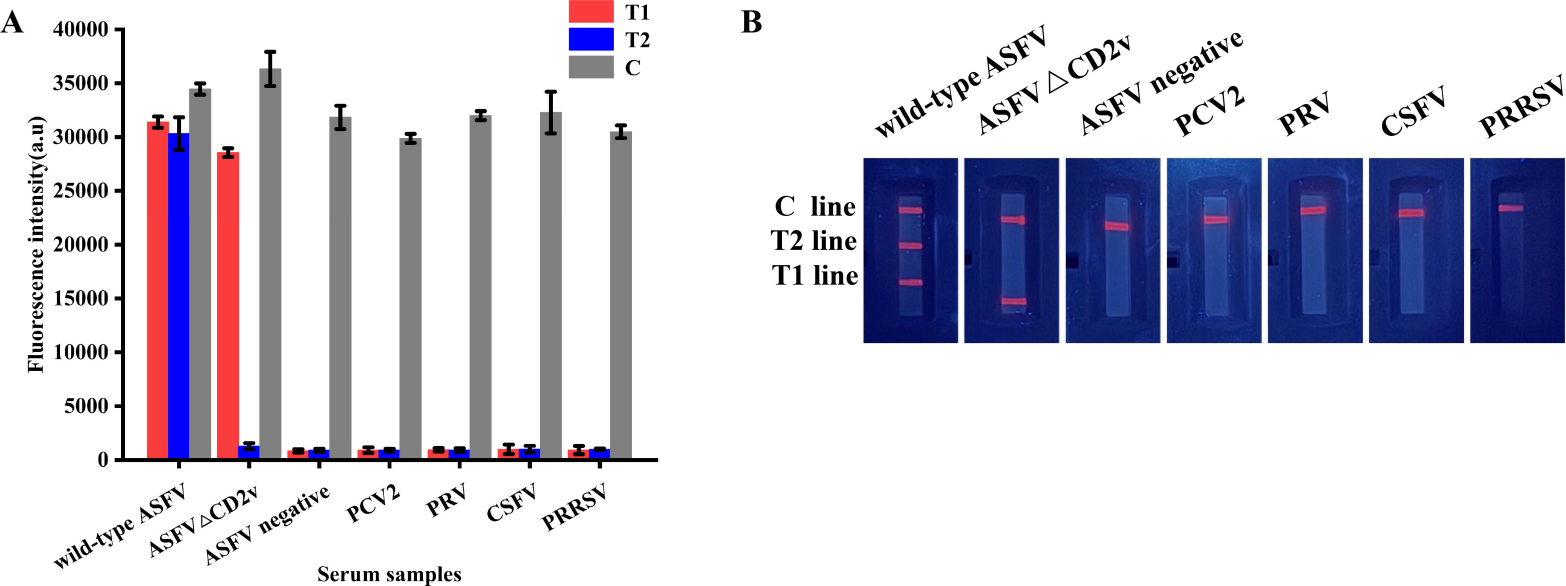
Analytical specificity of QDs dual ICS. (**A**) FI_T1_, FI_T2_, and FI_C_ with different positive serum of swine diseases antibodies by fluorescence immunoassay analyzer. (**B**) Image of T1, T2, and C line with different positive serum of swine diseases antibodies by portable UV lamp.

The analytical specificity of QDs dual ICS was verified by detecting the positive serum of swine disease antibodies. The results showed that, except for the positive serum of wild-type ASFV that displayed three bands at T1, T2, and C lines, and the positive serum of ASFVΔCD2v, which displayed two bands at T1 and C lines, all other positive sera of swine diseases detected in this study showed only one band at the C line (Fig. 10A and B). This indicated that the QDs dual ICS had no cross-reactions with the positive sera of other swine diseases, thereby demonstrating excellent analytical specificity.

#### Repeatability and reproducibility of QDs dual ICS

**Fig 11:**
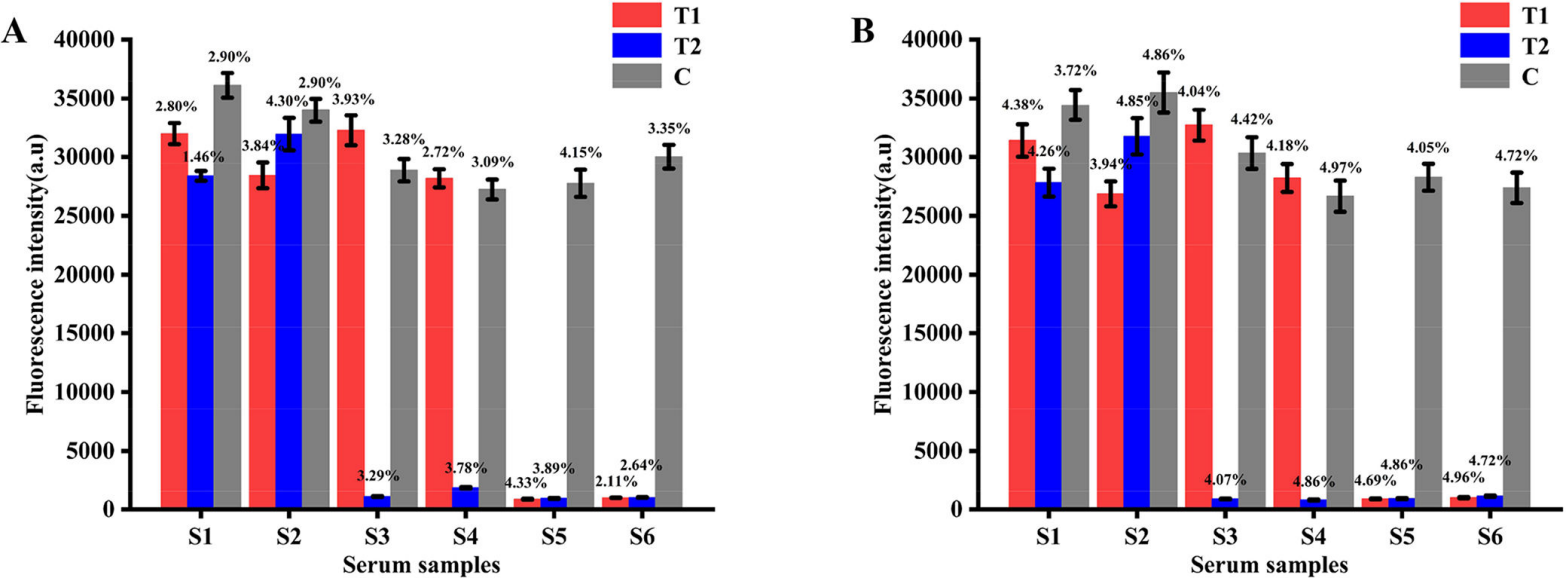
Repeatability and reproducibility of QDs dual ICS. Intra-batch CV (**A**) and inter-batch CV (**B**) of values FI_T1_, FI_T2_, and FI_C_ with serum samples in different states by fluorescence immunoassay analyzer.

The repeatability and reproducibility of the QDs dual ICS were evaluated by detecting the serum samples in different states. The results showed that the intra-batch CV (Fig. 11A) and inter-batch CV (Fig. 11B) values of FI_T1_, FI_T2_, and FI_C_ were all less than 5%, demonstrating excellent repeatability and reproducibility.

#### Stability of QDs dual ICS

**Fig 12:**
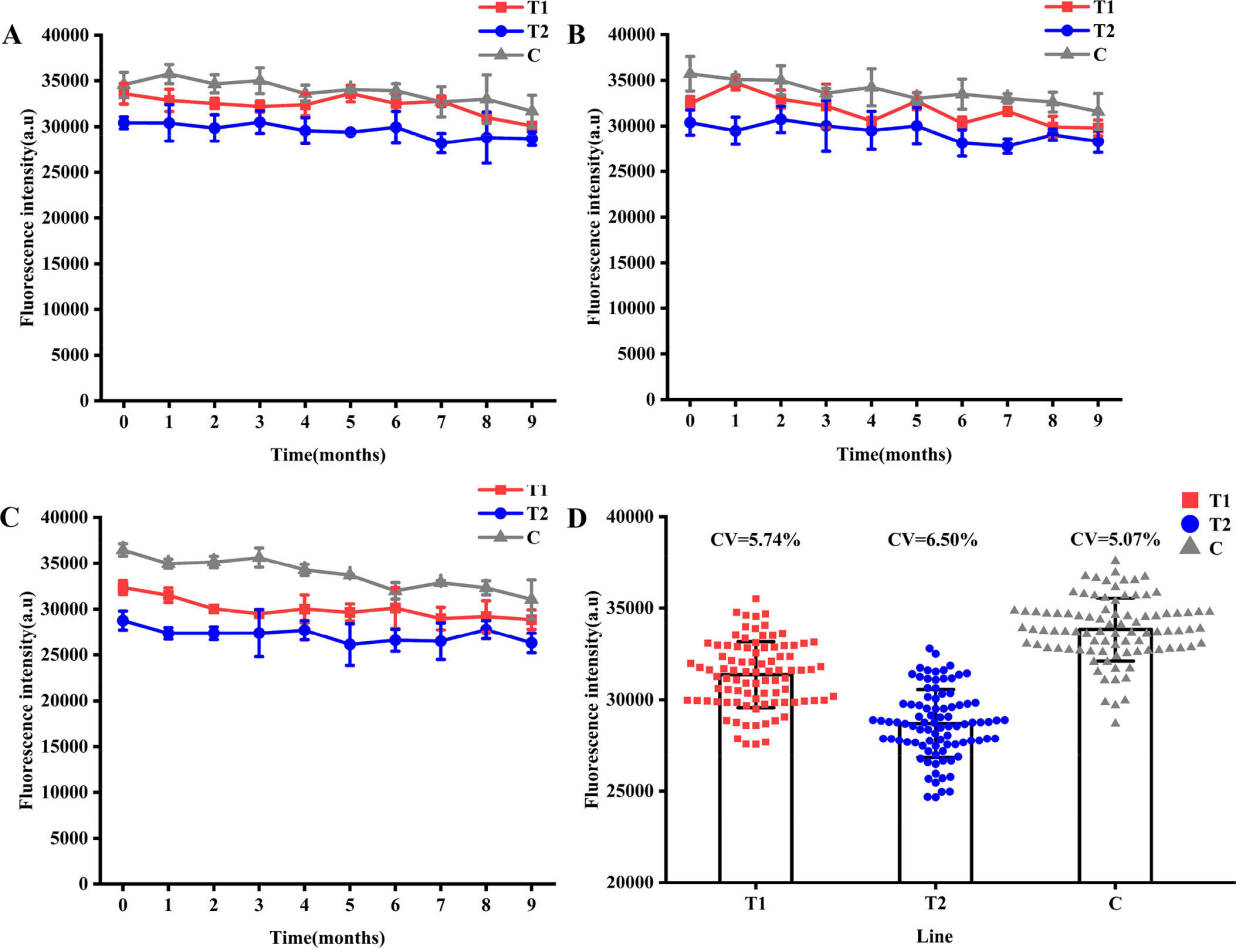
Stability of QDs dual ICS. FI_T1_, FI_T2_, and FI_C_ with serum sample detected by the same batch of QDs dual ICS stored at 4°C (**A**), 25°C (**B**), and 37°C (**C**) for 0, 1, 2, 3, 4, 5, 6, 7, 8, and 9 months. (**D**) CV values of FI_T1_, FI_T2_, and FI_C_ with serum sample detected by the same batch of QDs dual ICS stored at 4, 25 and 37°C for 0, 1, 2, 3, 4, 5, 6, 7, 8, and 9 months.

The stability of the QDs dual ICS was confirmed by detecting serum samples using the same batch of QDs dual ICS stored at 4°C, 25°C, and 37°C for 0, 1, 2, 3, 4, 5, 6, 7, 8, and 9 months. The FI results indicated that FI_T1_, FI_T2_, and FI_C_ of the QDs dual ICS stored at 4°C (Fig. 12A), 25°C (Fig. 12B), and 37°C (Fig. 12C) stabilized over time. CV analysis demonstrated that the CV values for FI_T1_, FI_T2_, and FI_C_ of the QDs dual ICS stored at 4°C, 25°C, and 37°C for 0, 1, 2, 3, 4, 5, 6, 7, 8, and 9 months were consistently below 7% (Fig. 12D). These findings confirm the long-term storage stability of the QDs dual ICS prepared in this study at various temperatures.

#### Detection of clinical samples

**Fig 13:**
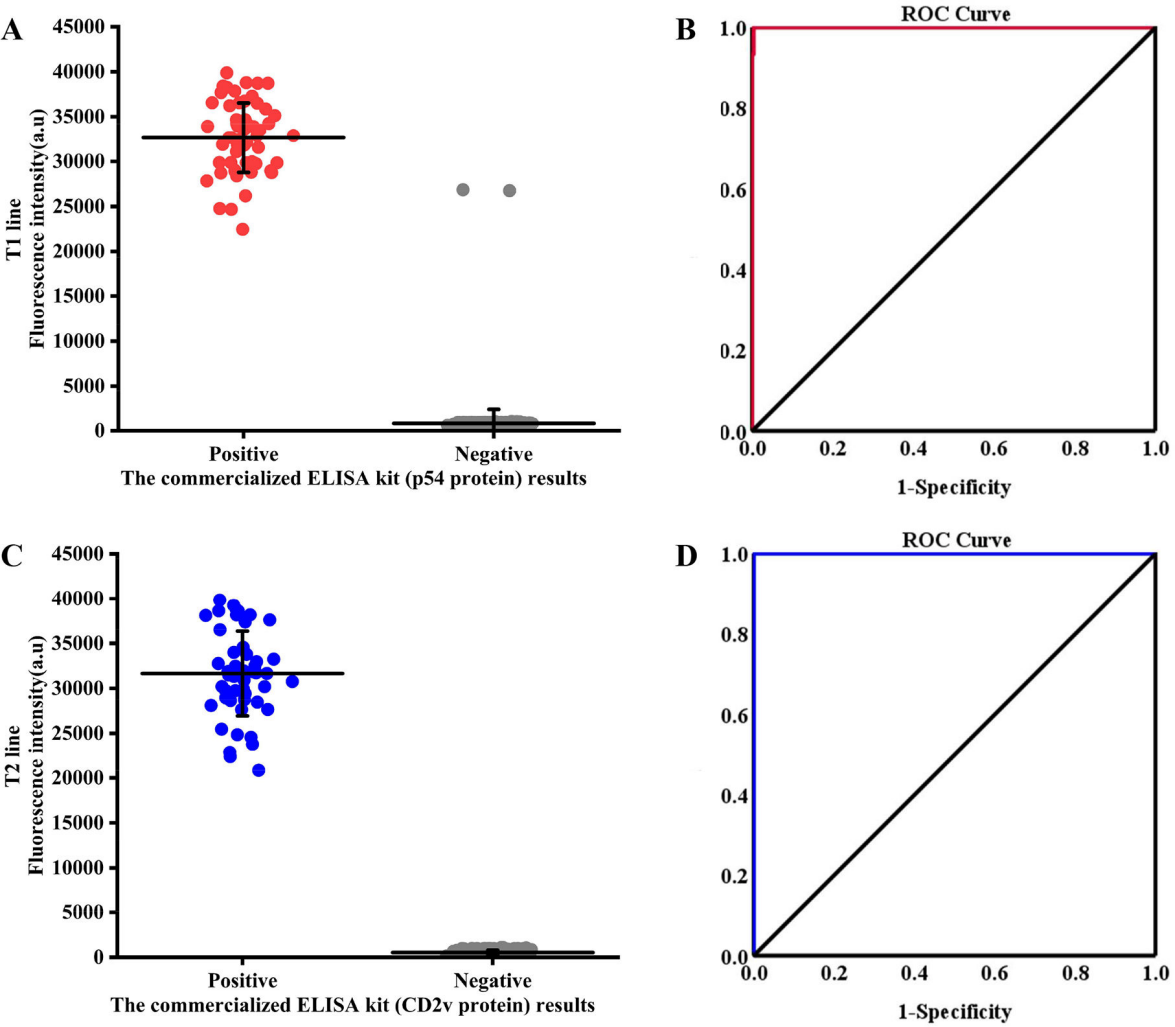
Detection of clinical samples. FI_T1_ (**A**) and ROC analysis (**B**) of QDs dual ICS (T1 line) and the commercial ELISA kit (p54 protein). FI_T2_ (**C**) and ROC analysis (**D**) of QDs dual ICS (T2 line) and the commercial ELISA kit (CD2v protein).

A total of 589 clinical samples were analyzed using commercial ASFV antibody ELISA kits and QDs dual ICS to evaluate the applicability of QDs dual ICS. The commercial ASFV antibody ELISA kit based on p54 protein (Kernel, USA) detected 60 samples as positive and 529 samples as negative (Fig. 13A), and the commercial ASFV antibody ELISA kit based on CD2v protein (Anheal, China) detected 52 samples as positive and 537 samples as negative (Fig. 13C). The QDs dual ICS detection results showed a total of 62 T1 bands (Fig. 13A), 52 T2 bands (Fig. 13C), and 589 C bands, indicating that 62 samples were positive and 527 samples were negative for ASFV antibodies. Among the 62 positive samples detected, 52 samples were positive for wild-type ASFV and 10 samples were positive for ASFVΔCD2V. Compared to the ELISA results, the QDs dual ICS demonstrated that only two samples exhibited different results on the T1 line, achieving an accuracy of 99.660% [(60 + 527) / 589 × 100%] (Table 1). By contrast, the T2 line achieved a perfect accuracy of 100% [(52 + 537)/589 × 100%] (Table 1). Based on the above results, the receiver operating characteristic (ROC) curves for p54 and CD2v proteins were constructed. Through ROC curve analysis, it was found that the T1 line of QDs dual ICS showed 100% sensitivity and 99.622% specificity compared to the commercial ELISA kit based on p54 protein, and the area under the curve (AUC) was close to 1 (Fig. 13B). Additionally, the T2 line of QDs dual ICS demonstrated 100% sensitivity and specificity compared to the commercial ELISA kit based on CD2v protein, with an AUC value of 1 (Fig. 13D). Further analysis indicated that the kappa values corresponding to the T1 and T2 lines were 0.981 and 1.000, respectively, which demonstrated the high consistency of the detection results.

**TABLE 1:** Comparison of the results of QDs dual ICS with the commercial ELISA kits

Detection methods and determination indices		The T1 line of QDs dual ICS		Total
		Positive	Negative	
The commercial ELISA kit based on p54 protein	Positive	60	0	60
	Negative	2	527	529
Total		62	527	589

Detection methods and determination indices		The T2 line of QDs dual ICS		Total
		Positive	Negative	
The commercial ELISA kit based on CD2v protein	Positive	52	0	52
	Negative	0	537	537
Total		52	537	589

## DISCUSSION

ASFV poses a substantial threat to the pig farming industry and results in significant economic losses (1, 2). In the absence of commercial vaccines or effective treatments, the control and prevention of ASFV primarily depend on timely and accurate diagnostic methods as well as stringent biosecurity measures. Among the diagnostic methods for ASFV, PCR and real-time are essential for nucleic acid detection. However, with the emergence and widespread dissemination of ASFVΔCD2v, the epidemiological landscape of ASFV has become increasingly complex. In pigs infected with ASFVΔCD2v, viral loads tended to be relatively low, whereas antibody persistence was prolonged. Relying exclusively on nucleic acid detection may not suffice for timely identification of ASFV infection status. Consequently, monitoring ASFV-specific antibodies provides essential insights into the status of pig infections.

Currently, the main methods for detecting ASFV antibodies are ELISA and ICS. Although ELISA can effectively detect ASFV-specific antibodies, its complex procedures, reliance on trained personnel, and inability to perform POCT limit its application. By contrast, ICS is characterized by its operational simplicity, does not require specialized expertise, and enables rapid POCT within approximately ten minutes. It is recognized as a key method for rapid disease diagnosis. Among ICS, CG ICS is extensively utilized for the detection of ASFV antibodies. Zhu established a double-antigen sandwich lateral flow assay based on the gold nanoparticle-labeled ASFV major capsid protein p72 (45). Wan developed a CG ICS for the detection of antibodies against ASFV p30 and p72 proteins using a 1:1 mixture of gold-labeled p30 and p72 probes as gold-labeled antigens (46). Liu developed an ASFV antibody test strip based on recombinant ASFV p30 protein and its monoclonal antibody (47). All of these methods can detect ASFV antibodies within 5–10 min and exhibit excellent specificity. However, despite enabling rapid POCT, these methods require further improvement in sensitivity. Therefore, ICS based on fluorescent microspheres has been developed to improve the detection performance. Li developed a fluorescence immunochromatographic test strip for the specific detection of ASFV antibodies using truncated p54 protein as the antigen and combined it with Eu-doped fluorescent microspheres as tracers (17). This strip can detect 1:1,280 diluted positive serum and demonstrates excellent specificity. Zhou developed an ASFV antibody test strip by labeling pp62 with QDs, achieving a positive detection limit of 1:10^6^ (39). Liu conjugated QDs with the ASFV p72 protein and developed a fluorescence immunochromatographic method for detecting ASFV-specific antibodies (38). This method achieved detection within 15 min, with a detection limit of up to 1:10^7^ diluted positive serum samples. However, although the aforementioned methods showed significantly improved sensitivity compared with CG ICS, they are incapable of distinguishing between ASFVΔCD2v and wild-type ASFV infections for differential diagnosis. Therefore, it is necessary to develop a rapid, highly sensitive, and highly specific ICS to detect ASFV antibodies and differentiate between ASFVΔCD2v and wild-type ASFV infections.

In this study, QDs were employed as tracers to develop a highly sensitive, specific, and stable ICS based on p54 and CD2v proteins. These QDs dual ICS are not only suitable for POCT of ASFV antibodies but are also capable of effectively distinguishing between ASFVΔCD2v and wild-type ASFV infection. The proteins used as antigens in serological methods for antibody detection should exhibit high antigenicity. The p54 protein serves as a major structural component of ASFV and can induce neutralizing antibodies during the early stages of ASFV infection. This protein exhibits strong immunogenicity and antigenicity, and is relatively conserved across different genotypes and strains (23–25). It is an ideal antigen for serological diagnoses. CD2v plays a critical role in host immune regulation and eliciting protective immune responses (28, 29). The absence of CD2v protein expression is the main characteristic of the ASFVΔCD2v strain and naturally occurring low virulence genotype I ASFV strains; therefore, CD2v protein is also an important indicator for differentiating wild-type ASFV from ASFVΔCD2v infections (1, 12–15). In the QDs dual ICS developed in this study, the p54 protein functions as the T1 line, CD2v protein as the T2 line, and anti-SPA mAb as the C line. These QDs dual ICS are capable of simultaneously performing the specific detection of antibodies against the p54 and CD2v proteins of ASFV. To ensure the optimal performance of the QDs dual ICS, p54 and CD2v proteins were successfully expressed at high levels via a prokaryotic expression system. To improve the detection efficiency of the test strip, the preparation conditions for the QDs dual ICS were systematically optimized. The results demonstrated that the optimal pH value for the conjugation of QDs with SPA was 7.0, and the optimal concentration for conjugating SPA with QDs was 0.25 µg/µL. Based on these findings, the coating concentrations of the T1, T2, and C lines were further refined. The optimal coating concentrations for the p54 protein, CD2v protein, and anti-SPA mAb were determined to be 2, 1.5, and 1.5 mg/mL, respectively. Additionally, the optimal sample dilution was determined to be 1:100 and the optimal immunoreaction time was 15 min. The results of the specificity test revealed that the QDs dual ICS exhibited no cross-reactivity with other positive sera of swine disease antibodies, thereby demonstrating exceptional specificity. The sensitivity test further indicated that the detection limit of the test strip was 1:10^6^, which is comparable to that of the test strip developed by Zhou (39). The QDs dual ICS demonstrated superior sensitivity compared with both the CG ICS reported in the literature and the Eu-doped fluorescent microspheres europium ICS developed by Li (17, 46, 47). Repeatability, reproducibility, and stability were systematically evaluated to ensure their clinical applicability and facilitate the batch production of the QDs dual ICS. Repeatability and reproducibility tests showed that the CV of FI_T1_, FI_T2_, and FI_C_ were below 5%, demonstrating excellent repeatability and reproducibility. The stability test results demonstrated that the CV of FI_T1_, FI_T2_, and FI_C_ during serum detection remained below 7% after storage at 4°C, 25°C, and 37°C for varying durations. The QDs dual ICS exhibited excellent stability under diverse storage conditions. In addition, upon evaluating the detection accuracy of the QDs dual ICS by detecting clinical samples, a significantly enhanced performance was observed compared to commercial ELISA kits. Specifically, the T1 line demonstrated a detection accuracy of 99.66% [(60 + 527)/589 × 100%] for the p54 protein with both 100% sensitivity and 99.622% specificity. Meanwhile, the T2 line achieved a detection accuracy of 100% [(52 + 537)/589 × 100%] for the CD2v protein, achieving a perfect sensitivity and specificity of 100%. Notably, this QDs dual ICS exhibited the capability of precisely detecting eight ASFVΔCD2v samples, a performance unmatched by other test strips.

In conclusion, a QDs dual ICS based on the p54 and CD2v proteins was developed. It can distinguish between naturally occurring low virulence genotype II ASFVΔCD2v and high virulence genotype II wild-type ASFV infection while exhibiting rapid, sensitive, and accurate POCT capabilities. Because the genotype I/II recombinant ASFV also expresses CD2v protein while the low virulence genotype I does not. The current developed QDs dual ICS can also be used to distinguish between genotype I/II recombinant ASFV and low virulence genotype I ASFV or II ASFVΔCD2v, as well as high virulence genotype II wild-type ASFV and low virulence genotype I ASFV. However, although the QDs dual ICS can distinguish between high virulence (CD2v expression) and low virulence (CD2v non-expression) ASFV infection. When the samples were shown positive on both the T1 and T2-lines, it could not distinguish between genotype II wild-type ASFV and genotype I/II recombinant ASFV, because they can both express CD2v protein. Similarly, it can also not distinguish between CD2v non-expression low virulence genotype II ASFVΔCD2v and genotype I ASFV. Therefore, future efforts should focus on developing antibody detection methods capable of more precise discrimination among various epidemic strains, thereby better supporting ASFV prevention and control practices. Furthermore, the scope of sample collection in this study was limited and did not encompass samples from all ASFV-affected regions in China or globally. Future research should prioritize enhancing the collection and validation of samples from diverse regions and various ASFV genotypes to further improve the universality and reliability of this method.

## Data Availability

The data sets generated during the current study are available from the corresponding author upon reasonable request.
